# A Haptic Sleeve as a Method of Mechanotactile Feedback Restoration for Myoelectric Hand Prosthesis Users

**DOI:** 10.3389/fresc.2022.806479

**Published:** 2022-04-25

**Authors:** Violet R. Borkowska, Alistair McConnell, Sethu Vijayakumar, Adam Stokes, Aidan D. Roche

**Affiliations:** ^1^Edinburgh Medical School, College of Medicine and Veterinary Medicine, The University of Edinburgh, Edinburgh, United Kingdom; ^2^Scottish Microelectronics Centre, Institute for Integrated Micro and Nano Systems, School of Engineering, The University of Edinburgh, Edinburgh, United Kingdom; ^3^School of Informatics, Bayes Centre, The University of Edinburgh, Edinburgh, United Kingdom; ^4^College of Medicine and Veterinary Medicine, The Queen's Medical Research Institute, The University of Edinburgh, Edinburgh, United Kingdom; ^5^Department of Plastic Surgery, National Healthcare System Lothian, Edinburgh, United Kingdom

**Keywords:** haptic, mechanotactile, sensory feedback, sensory restoration, prosthetic, prosthesis, hand, upper limb

## Abstract

Current myoelectric upper limb prostheses do not restore sensory feedback, impairing fine motor control. Mechanotactile feedback restoration with a haptic sleeve may rectify this problem. This randomised crossover within-participant controlled study aimed to assess a prototype haptic sleeve's effect on routine grasping tasks performed by eight able-bodied participants. Each participant completed 15 repetitions of the three tasks: Task 1—normal grasp, Task 2—strong grasp and Task 3—weak grasp, using visual, haptic, or combined feedback All data were collected in April 2021 in the Scottish Microelectronics Centre, Edinburgh, UK. Combined feedback correlated with significantly higher grasp success rates compared to the vision alone in Task 1 (*p* < 0.0001), Task 2 (*p* = 0.0057), and Task 3 (*p* = 0.0170). Similarly, haptic feedback was associated with significantly higher grasp success rates compared to vision in Task 1 (*p* < 0.0001) and Task 2 (*p* = 0.0015). Combined feedback correlated with significantly lower energy expenditure compared to visual feedback in Task 1 (*p* < 0.0001) and Task 3 (*p* = 0.0003). Likewise, haptic feedback was associated with significantly lower energy expenditure compared to the visual feedback in Task 1 (*p* < 0.0001), Task 2 (*p* < 0.0001), and Task 3 (*p* < 0.0001). These results suggest that mechanotactile feedback provided by the haptic sleeve effectively augments grasping and reduces its energy expenditure.

## Introduction

According to the closed loop theory of motor control, movement of a healthy human hand is governed by co-dependant feedforward muscle control and sensory feedback (1). Based on the latter, feedforward muscle control is adjusted to achieve economy of movement and the lowest possible metabolic energy expenditure (2), therefore closing the loop. When limb loss occurs, the loop of motor control becomes disrupted. The feedforward component of the loop may be partially restored with myoelectric prostheses (3). However, these devices do not restore sensory feedback, leaving the loop of motor control open (4). As a result, prosthetic users only have uncertain feedforward control at their disposal (5), making them unable to perceive tactile properties of handled objects and experience diminished motor control (6). They cannot feel their prosthetic grip force, leading to application of excessive force (resulting in excessive energy expenditure and muscle fatigue) and crushing of handled objects (7–9). In order to achieve satisfactory prosthetic performance, users heavily rely on visual feedback, which in turn increases cognitive load (10, 11). For these reasons, prosthesis embodiment remains poor, as reflected in the prosthesis abandonment rate of 40% (12).

Considering the above, it is unsurprising that most amputees agree restoring sensory feedback is as important as restoring feedforward muscle control (7, 13–15). Restoring tactile feedback, a type of sensory feedback, is especially promising. It has been shown to not only significantly improve grasp success rate (16–18) but also significantly decrease grip force (19–22). Restoring tactile feedback is also predicted to reduce prosthesis abandonment rate (23), providing a strong rationale for development of tactile feedback modalities.

So far, invasive and non-invasive tactile feedback modalities have been developed (24). Invasive modalities, such as targeted sensory re-innervation, direct peripheral nervous system stimulation and central nervous system stimulation, are promising due to their potential to elicit near-natural touch sensations (25–27). However, their clinical utility remains challenging (28). They carry a number of risks, such as nerve damage (29) and paraesthesia (30), have been tested on a limited number of volunteers and face much reluctance from amputees (31).

Non-invasive modalities, such as vibrotactile, electrotactile and mechanotactile feedback systems (28) are comparatively better characterised and constitute a more acceptable alternative as they require no surgical interventions (31). Yet, non-invasive modalities are not without their caveats. The main criticism of vibrotactile and electrotactile feedbacks is centred around their dissimilarity to endogenous tactile feedback, making them difficult to understand. Both are discontinuous (composed of discrete vibration or electric current bursts) and modality mismatched (vibrations or electric currents felt on the skin usually encode grip pressure), contrary to biological feedback (32). In contrast, mechanotactile feedback is both continuous and modality matched (pressure applied to skin encodes grip pressure). As such, it mimics natural tactile feedback, making the artificial feedback more intuitive to understand (32, 33). However, these advantages are balanced out by mechanotactile devices being larger, heavier, and of greater energy demands than their electrotactile and vibrotactile counterparts, inhibiting their development (34).

Disadvantages associated with invasive and non-invasive tactile feedback modalities contribute to their clinical and commercial unavailability. Mechanotactile feedback, as the only non-invasive modality providing continuous and modality matched feedback, seems to have an underdeveloped potential. Hence, research into how its current caveats can be resolved is warranted.

This study aimed to test the utility of a new mechanotactile feedback restoration device, a prototype haptic sleeve. Haptic sleeves are sleeve-shaped, variable compression devices which have so far demonstrated utility in robot-assisted surgery (35), social touch mimicking (36), and virtual reality enhancement (37). They are lightweight and thin, addressing the problems of heaviness and large size characteristic of contemporary mechanotactile feedback devices. While haptic band devices have been developed to provide sensory feedback in rehabilitation robotics, they have not uniformly been integrated into a prosthetic sleeve which is an integral part of the socket (38–41). Where a pneumatic device has been integrated into the socket, it has been at a discrete point instead of providing distributed sensory feedback across the residual limb (42). Our study demonstrates a soft socket that integrates haptic feedback across its inner surface whilst being capable of supporting the terminal device without need for any additional material.

The primary aim of the study was to be achieved by assessing the impact of the haptic sleeve on grasp success rate and energy expenditure of grasping. Grasp success rate was chosen as it is a simple, concrete metric which is in wide use in tactile feedback restoration studies (16–18). However, it is an indirect measure of tactile feedback impact on motor control, making it difficult to speculate about a cause-and-effect relationship. Therefore, changes in energy expenditure of grasping were recorded, too, as they are a more direct and robust basis for establishing a causal link between feedback restoration and improvement in outcomes (2). It was hypothesised that using the haptic sleeve will result in higher grasp success rate and reduced energy expenditure of grasping.

## Materials and Methods

### Design of the Haptic Sleeve

#### Wearable Sleeve

The prototype haptic sleeve used in the experiments was designed by the research team and manufactured by Koalaa Prostheses (London, UK) and can be seen in Supplementary Figure 1. Once mounted, the sleeve extended from the participant's proximal forearm to their wrist, allowing for a secure and comfortable fit while leaving enough space for the EMG electrode placement immediately distal to the elbow joint. The device was designed to compress the forearm proportionally to the pressure detected at prosthetic fingertips, thus delivering continuous mechanotactile feedback. To execute this function, the sleeve had a small motor (RS Pro 951D, RS Components, London, UK) mounted on its lateral side, as well as a pulley system with a thread wrapped around the sleeve equidistantly several times. Clockwise rotation of the motor resulted in winding of the thread around the sleeve, tightening it and therefore compressing the user's forearm. Anticlockwise rotation of the motor unwound the strings, untightening the sleeve and reducing the compression.

#### Electrical Design

To provide all the required analogue and digital inputs and outputs to the system, an Arduino Uno REV3 microprocessor (Arduino, Massachusetts, USA) was used. Connected to the microprocessor via a protoboard were two force-sensing resistors (FSRs) (Interlink Electronics FSR400, California, USA), three separate push button switches, a L298N 2A motor driver (HandsOn Tech, Johor, Malaysia), adjustable power supply (30V 3A Tenma 72-2540) and a Y-bridge. The electronic circuit obtained functioned to detect force applied at FSRs on prosthetic fingertips and translate it into rotation of the haptic sleeve's motor. It also recorded the EMG signals whilst they were used for the myoelectric prosthesis control. Simultaneous EMG signal recording and use was enabled by the Y-bridge which split the EMG signals from the electrodes into two channels. One channel connected to the prosthesis, facilitating myoelectric control, while the other channel connected to the microprocessor, allowing signal recording.

#### Software/Hardware Interface

The microprocessor was programmed using the Arduino Integrated Development Environment (Arduino, Massachusetts, USA). Its main functions were to record and save experimental readings, as well as interpret the FSR readings by comparing their current averaged force readings to their previous averaged value. If the new value was greater than the previous one, the motor engaged for 0.1 s at the speed proportional to the new value, tightening the sleeve. However, if the new average force was smaller than the previous one, the motor retained its current position. In this way, the haptic sleeve could provide continuous, proportional mechanotactile feedback. The Pulse Width Modulation (PWM) of the motor at time *t* is defined by the following equations:


(1) $$\begin{array}{rcl} {\bar{F}}_{t} & = & \left(\frac{F_{Index}+\;F_{Thumb}}{2}\right) \end{array}$$

(2) $$PWM\;=\;\{\begin{array}{c} 0,\;\\ \frac{{\bar{F}}_{t}}{F_{MAX}}\;\times 100\;\times \;PWM_{Max}, \end{array}\begin{array}{c} F_{t}\;\leq \;F_{t-1}\\ F_{t}>F_{t-1} \end{array}$$

Where ${\bar{F}}_{t}$ is the new average force, F_t−1_ equals the previous average force, F_Index_ is equal to the force from the index finger sensor, F_Thumb_ is the force from the thumb sensor, *F*_*MAX*_ equals the maximum force of the sensor and *PWM*_*Max*_ is the maximum PWM value.

The minimum force applied by the device was 0N, while the maximum force that the sleeve could generate was 5.1N.

All data recorded by the microprocessor during the experimental attempts was transferred to a PC via a USB serial cable and read and displayed in real-time via PuTTY application. Once each run was completed the application was closed and the data saved as a .txt file.

### Ethical Considerations

The study was approved by the Informatics Research Ethics Board of the University of Edinburgh (2019/23785). Written consent was obtained from all participants prior to any experimentation.

### Participant Selection

Participant inclusion criteria were being able-bodied and over the age of eighteen. Exclusion criteria were having a musculoskeletal disorder or prior experience with myoelectric control.

### Experimental Setup

**Figure 1:**
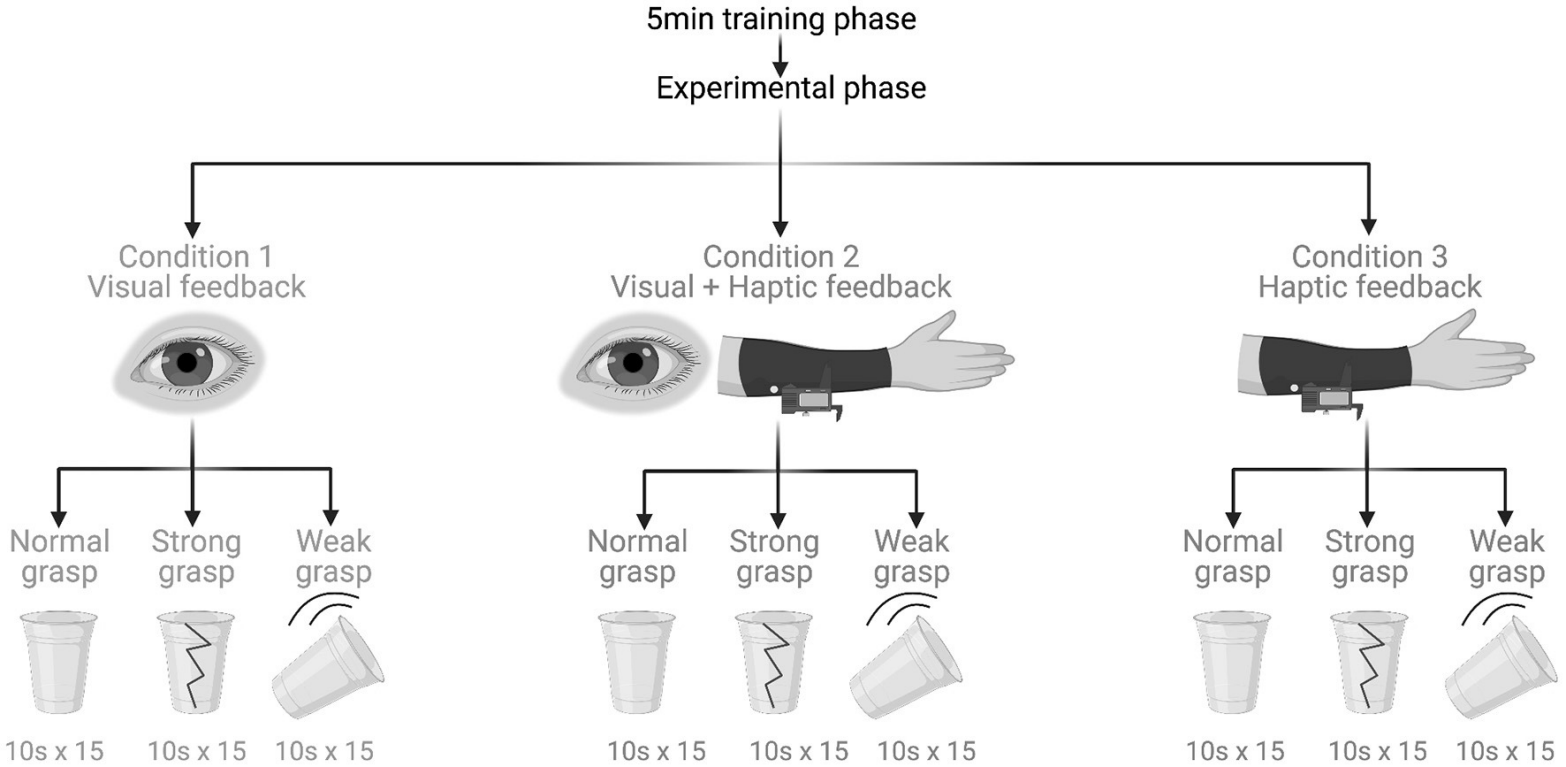
Experimental protocol and tasks. Created with BioRender.com.

Figure 1 and Supplementary Figure 1 depict the experimental setup. The electronic circuit, the Y-bridge and the biological hand were all positioned on the table. The myoelectric prosthetic hand was clamped on the edge of the table so that the experimental object it grasped was unsupported. The prosthesis used in this study was a six degree-of-freedom Nexus Hand (COVVI, Leeds, UK). However, only one degree of freedom was used as this allowed optimal replication of the grasping motion. Participants operated the prosthesis using two 50 Hz Össur surface electrodes with built-in EMG signal amplifiers and philtres (Össur, Reykjavik, Iceland). One electrode was adhered to the skin over the forearm extensor digitorum communis muscle group whist the other over the flexor digitorum superficial is muscle group. The experimental object was a 295 ml plastic tumbler cup. When the grasp force applied was >4.5N, the cup broke, producing a distinctive, loud noise. After each breakage, the cup was replaced with a new one.

### Experimental Protocol

**Figure 2:**
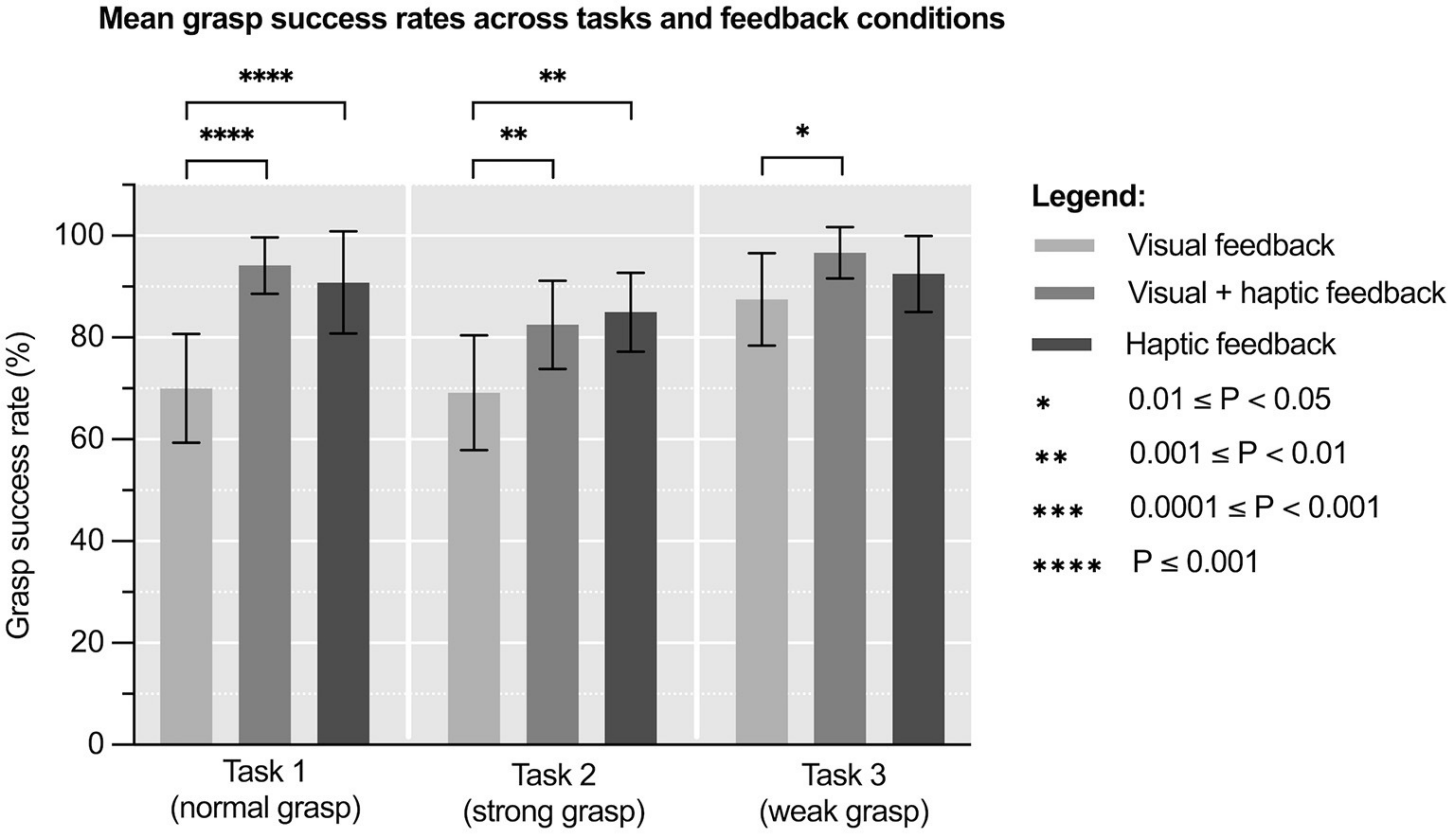
Mean grasp success rates (%) across tasks and feedback conditions. Error bars represent standard deviation. *N* = 8.

This study was a crossover randomised within-participant controlled trial. It began with a 5-min-long training phase consisting of guided familiarisation with myoelectric control. Next was the experimental phase made up of three experimental tasks that were performed under three feedback conditions (Figure 2). All participants completed all tasks under all conditions.

### Sensory Feedback Conditions

Under the *visual feedback condition*, the participants did not wear the haptic sleeve and had their vision unobstructed. Thus, it was employed as a control condition. Under the *visual plus haptic feedback condition*, participants could still see but they also received mechanotactile feedback through the haptic sleeve. Under the *haptic feedback condition*, participants still received mechanotactile feedback but this time their vision was disabled by a blindfold. Any incidental auditory feedback was attenuated with the use of white noise-emitting headphones that participants wore at all times.

### Experimental Tasks

Under each condition, participants had to perform fifteen 10-s-long repetitions of each task. In Task 1, *normal grasp*, the participants were instructed to grasp the experimental object with the myoelectric prosthesis so that the object neither breaks nor drops. Task 2, *strong grasp*, was the same as Task 1 but the instruction was to grasp the object as strongly as possible without breaking it. Task 3, *weak grasp*, was again the same as Task 1 but the command was to grasp the object as lightly as possible without dropping it. The purpose of the varying grasp strengths was to assess the impact of haptic feedback on grip force adjustment.

### Randomisation

Simple randomisation was performed to obtain a unique sequence of feedback conditions and tasks for each participant. The sequences were generated in Research Randomizer (Social Psychology Network, USA) and the participants were blinded to their allocated sequence. The aim was to reduce confounding of the results by learning effects.

### Outcome Measures

The primary outcome measure was grasp success rate, expressed as a percentage of successful attempts. A successful attempt was defined as neither breaking nor dropping the experimental object. The secondary outcome measure was energy expenditure of grasping, equal to the indefinite integral of the EMG curve.

### Sample Size Calculation

Sample size calculation was performed based on preliminary primary outcome results for the first four participants with a mean of 75% grasp success rate and standard deviation of 9.97 with visual feedback alone, compared to a mean 98.25% and standard deviation of 3.03 with haptic feedback alone. Glass' *delta* between these two groups demonstrated an effect size of 2.33. Using this effect size, with a power of 80% and two-sided α level of 0.05, a desired sample size of eight participants was calculated using G^*^Power 3.1 (HHU, Düsseldorf, Germany).

### Statistical Analysis

All statistical analyses were conducted in Prism 9.1.0 (GraphPad, California, USA). The threshold for statistical significance was adopted at *p* < 0.05. Shapiro-Wilk test of normality was performed first and showed all data were parametric. To determine if there was significance between groups, two-way analysis of variance (ANOVA) with repeated measures were conducted as there were two factors influencing the data (feedback condition and task). Partial eta squared ($\eta_{\text{p}}^{2}$) was calculated as an effect size measure of any significant results. *Post-hoc* Tukey's test was used to further characterise any statistical significance.

## Results

### Study Demographics

In total, eight volunteers were recruited between March and April 2021. All of them met inclusion criteria and none were excluded. Hence, all volunteers were randomised; they completed all experimental tasks and were included in the analyses. Supplementary Table 1 summarises their demographic characteristics. All experiments were conducted in April 2021 at the Scottish Microelectronics Centre, Edinburgh, UK.

### Grasp Success Rate

**Figure 3:**
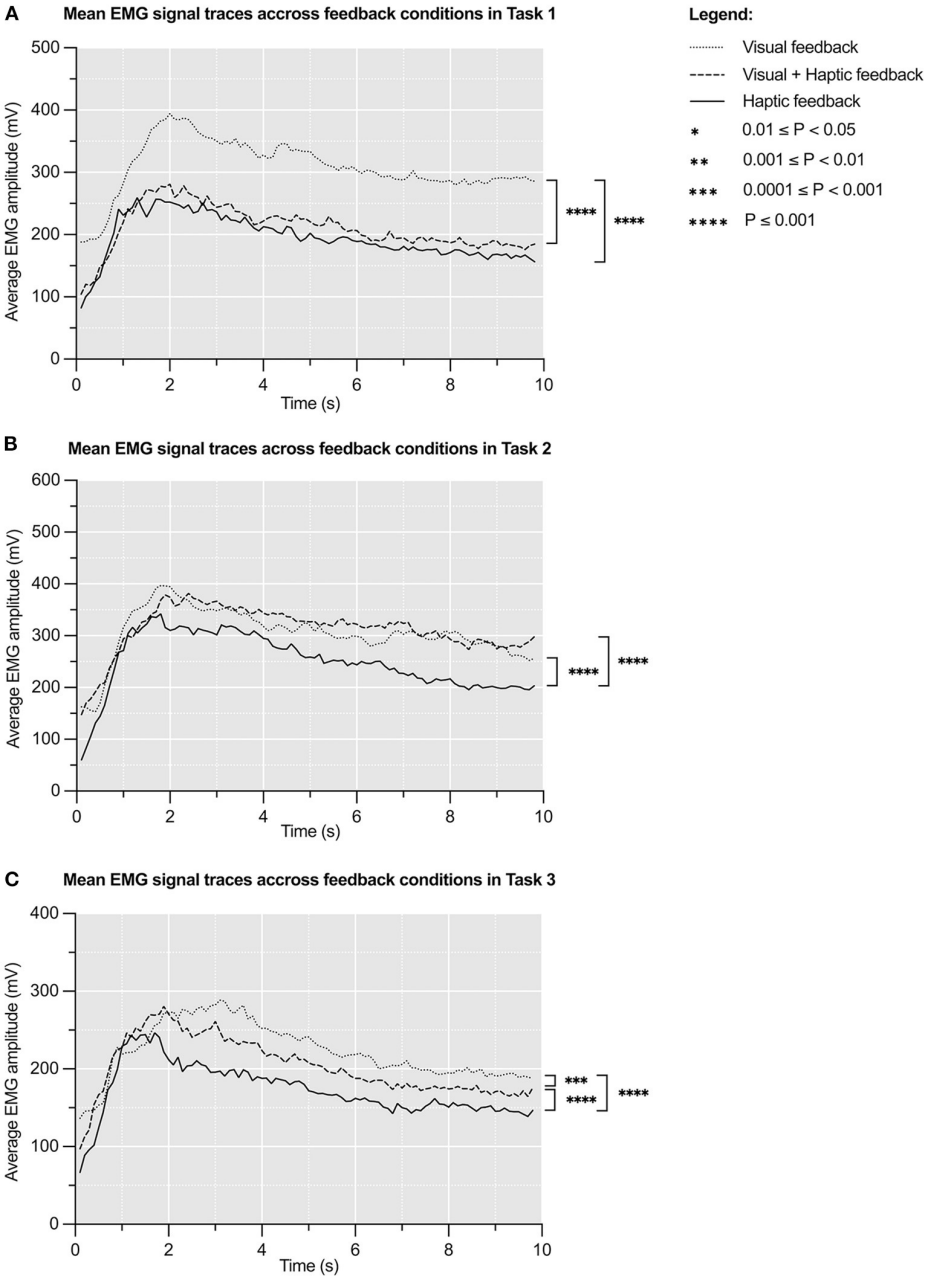
Mean EMG signal (mV) traces over 10s across feedback conditions in Task 1 **(A)**, Task 2 **(B)**, and Task 3 **(C)**. The initial large peak corresponds to grasping the experimental object and the latter plateau corresponds to sustained grip. *N* = 8.

Figure 3 and Supplementary Table 2 show mean grasp success rates across feedback conditions and tasks. One way repeated measures ANOVA of these results revealed significant variation in grasp success rates under different feedback conditions in Tasks 1 & 2, but not during Task 3: Task 1 [*F*_(1.91, 13.4)_ = 48.5, *p* < 0.0001], Task 2 [*F*_(1.25, 8.75)_ = 11.5, *p* = 0.006] and Task 3 [*F*_(1.99, 14.0)_ = 3.47, *p* = 0.06]. The effect size of this variation was $\eta_{\text{p}}^{2}$ = 0.87 (95% CI: 0.70–0.91) for Task 1, $\eta_{\text{p}}^{2}$ = 0.62 (95% CI: 0.24–0.73) for Task 2 and $\eta_{\text{p}}^{2}$ = 0.42 (95% CI: 0.04–0.59) for Task 3. This means that ~87, 62, and 42% of variability in the results, respectively, can be attributed to feedback condition.

A *post hoc* Tukey's test showed that the mean grasp success rates under *visual plus haptic feedback condition* were significantly higher compared to those under *visual feedback condition* in all tasks: Task 1 (+34.6%, *p* < 0.0001), Task 2 (+19.2%, *p* = 0.006), and Task 3 (+10.5%, *p* = 0.017). It also showed that the mean grasp success rates under *haptic feedback condition* were significantly higher compared to those under *visual feedback condition* in Task 1 (+29.7%, *p* < 0.0001) and Task 2 (+22.8%, *p* = 0.0015). No significant differences were found between mean grasp success rates under *visual plus haptic* and *haptic feedback condition* in any of the tasks.

### Energy Expenditure

**Figure 4:**
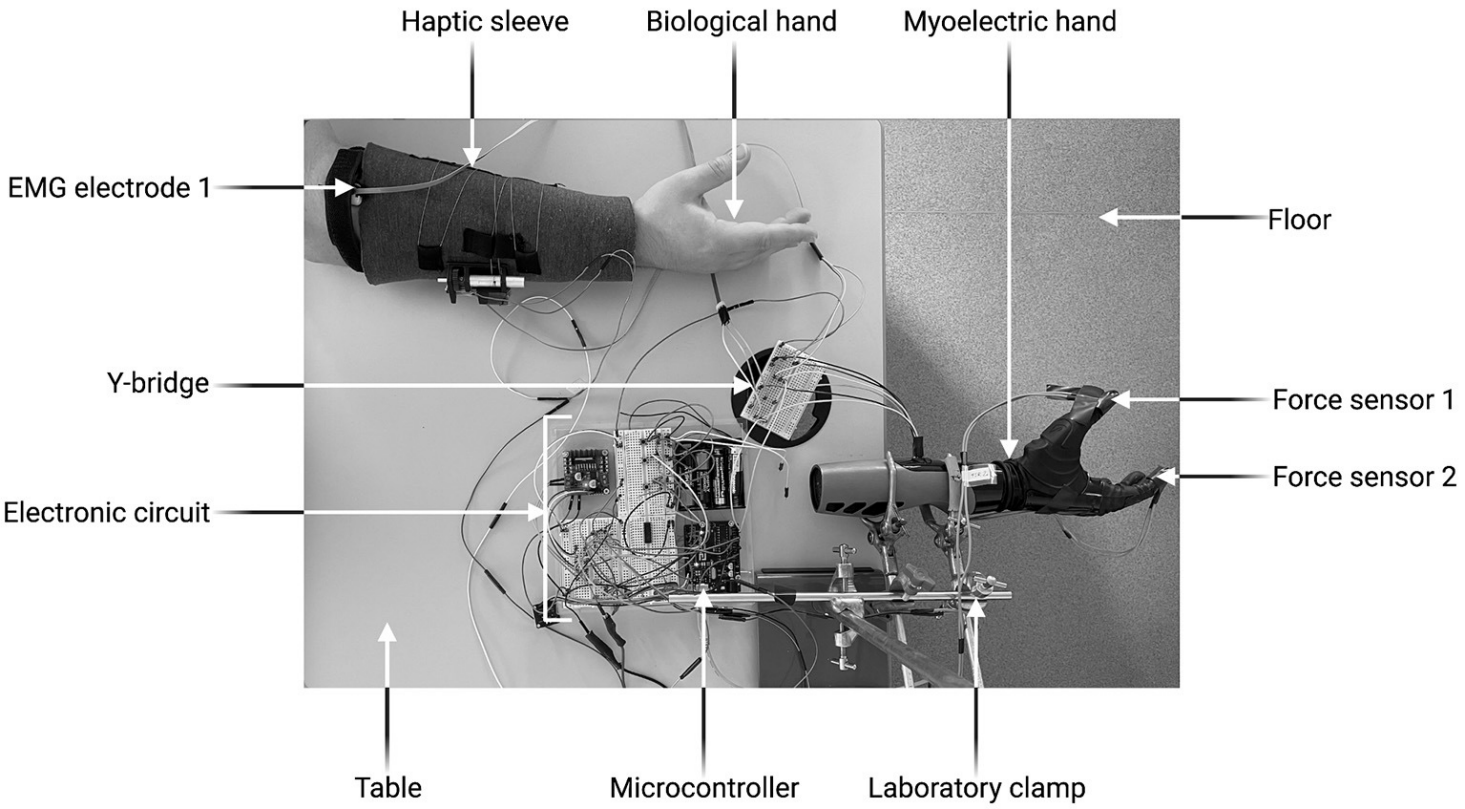
Experimental set-up. Created with BioRender.com.

Figure 4 represents mean EMG signal traces during all grasping attempts in respective tasks. These curves are timelines of participants' electromyographic activity (43). Mean areas under the EMG curves are presented in Supplementary Table 3. One way repeated measures ANOVA of these results revealed significant variation in mean areas under the EMG curves under different feedback conditions in all tasks: Task 1 [*F*_(1.31, 9.18)_ = 9.545, *p* < 0.009], Task 2 [*F*_(1.82, 12.8)_ = 6.36, *p* < 0.01), and Task 3 [*F*_(1.20, 8.41)_ = 9.51, *p* < 0.01]. The effect size of this variation was $\eta_{\text{p}}^{2}$ = 0.31 (95% CI: 0.24–0.37) for Task 1, $\eta_{\text{p}}^{2}$ = 0.12 (95% CI: 0.07–0.17) for Task 2 and $\eta_{\text{p}}^{2}$ = 0.24 (95% CI: 0.17–0.30) for Task 3. This means that feedback condition accounts for ~31, 12, and 24% of variability in the results across Task 1, 2, and 3, respectively.

A *post hoc* Tukey's test demonstrated that the *haptic feedback condition* was associated with significantly lower mean energy expenditure compared to the *visual feedback condition* in all tasks: Task 1 (−36.7%, *p* < 0.0001), Task 2 (−18.1%, *p* < 0.0001), and Task 3 (−22.4%, *p* < 0.0001). It also showed that the *visual plus haptic feedback condition* correlated with significantly lower mean energy expenditure compared to the *visual feedback condition* in Task 1 (−31.8%, *p* < 0.0001) and Task 3 (−8.7%, *p* = 0.0003).

## Discussion

### Key Findings and Interpretation

This study was the first to adapt and test a wearable sleeve that integrates haptic feedback across the prosthetic socket as a method of mechanotactile feedback restoration. It showed that using the device correlates with higher grasp success rate and lower muscle energy expenditure, which is consistent with the initial hypothesis. These findings are clinically relevant. Difficulty grasping and muscle fatigue are some of the most important factors contributing to high prosthesis abandonment rate (12). Minimising their impact could increase prostheses function, potentially elevating amputees' overall quality of life.

### Haptic Feedback Correlates With Higher Grasp Success Rate

Conditions including haptic feedback were found to be associated with significantly higher grasp success rates in all tasks (Figure 3). Additionally, feedback condition accounted for most variability in Task 1 and 2, suggesting a possible causative relationship. Correlation between non-invasive tactile feedback restoration and higher grasp success rates is well-established in bionic literature. Studies on electrotactile (16), vibrotactile (17), and mechanotactile (18) feedback all report the same trend. It is proposed that better grasp success rates result from participants utilising the feedback to better control the force they are applying (44). Another interesting finding was no significant difference in grasp success rates under *visual plus haptic* and *haptic feedback condition* in any of the tasks. There are two important implications to this. Firstly, it suggests that haptic feedback reduced participants' reliance on vision, a desirable phenomenon documented in other studies on tactile feedback (10, 11). Secondly, it might mean that the device's feedback delay is equal to, or even shorter than, visual feedback delay of 250 ms (45), making haptic feedback quick enough to be readily used (46).

### Haptic Feedback Correlates With Lower Energy Expenditure

Addition of haptic feedback was also noted to be correlated with significantly lower energy expenditure of grasping in all tasks, when the averaged EMG data was investigated across all trials (Figure 4). Moreover, it was estimated to be responsible for 12–31% of the variability in the results, suggesting a possible cause-and-effect relationship. It is challenging to compare this finding to existing literature as no previous study has calculated energy expenditure to assess sensory feedback restoration. Even studies that use EMG signals as a feedback modality (47, 48) do not report any outcomes in terms of energy expenditure. The reduction in energy expenditure demonstrated in this study can be explained by the closed loop motor control theory (1). According to the theory, sensory feedback is constantly used to achieve economy of movement and the lowest possible energy expenditure (2). Additionally, in prosthetic control systems, sensory feedback can partially rectify the inherent uncertainty of the feedforward myoelectric control, improving the overall motor control (5). Thus, it is possible that mechanotactile feedback provided by the sleeve closed participants' motor control loops effectively enough to allow for motor control and energy expenditure to be optimised.

### Strengths

#### Simultaneous EMG Signal Recording and Use

Muscle EMG traces are considered one of the most accurate methods of metabolic energy expenditure estimation for individual muscles and muscle groups (49). Measuring energy expenditure is of high importance in prosthetic research as it correlates with physical fatigue of prosthesis use (50). Fatigue, if excessive, leads to prosthesis abandonment (12, 51). Previous studies on tactile feedback restoration struggled to reliably record EMG signals due to inability to simultaneously record EMG signals and use them for myoelectric control (43). A Y-bridge, as used in this study, circumvents this issue by splitting the EMG signal registered by the electrodes into two independent channels. The technique is simple yet reliable and can be easily incorporated into future studies of this kind.

#### Experimental Sequence Randomisation

Haptic feedback use is characterised by a learning curve whereby functional outcomes improve with practise (52, 53). To reduce the confounding effects of learning on this study's results, simple randomisation of experimental task sequence was performed. Not only did this distribute the learning bias across feedback conditions and tasks, but also hindered learning by eliminating predictability (54). Despite each participant performing a unique sequence of tasks, significant changes were found in favour of haptic feedback, which corroborates the study's internal validity.

#### Adequate Sample Size and Power

The required sample size was met and therefore the study is 80% powered for the primary outcome to keep the probability of Type II error at 0.2 and, thus achieves a statistically sound balance with Type I error probability of 0.05 (55). However, as this study particularly looked at the impact of the tasks on EMG activity as a marker of energy expenditure, and there was limited previous data to calculate the sample size, our calculations were based on the pilot data of 4 participants. Consequently, while the significant results obtained in this investigation may reflect a true effect, further work with amputee participants should help corroborate these findings.

### Limitations

#### Lack of Amputee Participants

Due to the concurrent COVID-19 pandemic, recruiting amputee participants was made impossible. Considering the situation, the study was adapted to accommodate able-bodied participants. However, this was a suboptimal solution due to a number of significant differences between a residual limb and a healthy hand. During the amputation procedure, certain muscles and nerves are partially or completely removed, impeding subsequent EMG signal generation and making it more variable compared to able-bodied counterparts (56). Several changes may occur after the amputation, too. These include residual limb muscle atrophy, phantom limb pain or sensations, as well as contracture and neuromata formation (57). As a result, amputee participants may find it more difficult to not only generate EMG signals sufficient for myoelectric control, but also perceive and interpret the mechanotactile feedback provided by the haptic sleeve. Thus, the study's generalisability to amputee population is compromised.

#### Incomplete Natural Sensory Feedback Disablement

One common criticism of using able-bodied participants in studies assessing sensory feedback restoration is that these volunteers have their natural sensory feedback intact, which can confound the results (28). Although this study experimental setup meant that natural tactile feedback was negligible (a healthy hand did not touch the experimental object), participants still had their proprioception intact (they could move their biological hands). As a consequence, they were able to understand their hands' position even with their vision disabled, which could arguably result in better performance compared to amputees. To fully disable all sensory feedback, peripheral nerve blocks (58) or inflatable cuffs (59) have been used in similar studies in the field. However, the benefit of using these methods has to be balanced against their invasiveness and painfulness, as well as the requirement for additional ethical considerations.

#### Implications for Future Research and Clinical Practise

Future research should aim to further develop and test the haptic sleeve. Firstly, the device should be adapted for amputee use and assessed in the target user group. Additionally, the effects of the haptic sleeve need to be studied on a greater range of manipulative tasks. For that, established clinical tests assessing user performance in myoelectric control can be used, such as the Southampton Hand Assessment Procedure or the Action Research Arm Test (60). Any significant effects can later be studied over time to characterise the learning curve.

In order to successfully introduce the haptic sleeve onto the market and into clinical practise, it needs to be made portable. The current prototypic electronic circuit can be miniaturised into a compact control platform which will easily fit within the sleeve. If future studies corroborate clinical benefits of using the haptic sleeve, the device may become an integral element of the rehabilitation process after an upper limb amputation. Thanks to the sleeve, future amputees might be able to achieve better prosthetic function, which may translate into greater prosthesis embodiment, reduced phantom limb pain, enhanced quality of life and wider job opportunities.

## Conclusion

This study demonstrates that a haptic sleeve can be an effective tool for mechanotactile feedback restoration. Use of the device correlates with significantly higher grasp success rates and significantly lower energy expenditure of grasping in healthy volunteers. These findings are likely due to the haptic sleeve improving control of the applied force, decreasing reliance on vision and closing the motor control loop. Further research into the area is warranted and should focus on adapting the device for amputee use and improving its portability. With these enhancements, the haptic sleeve may help amputees recover more function and improve their quality of life.

## Data Availability Statement

The raw data supporting the conclusions of this article will be made available by the authors, without undue reservation.

## Author Contributions

AR conceived the study, with VB, AM, SV, and AS contributing to the design and implementation of the work. VB and AM collected the data and analysed the data with AR. All authors contributed to drafting and reviewing the manuscript before final approval for submission.

## Funding

The Academy of Medical Sciences supported this study through a Starter Grant for Clinical Lecturers (SGL021/1004) to the corresponding author.

## Conflict of Interest

The authors declare that the research was conducted in the absence of any commercial or financial relationships that could be construed as a potential conflict of interest.

## Publisher's Note

All claims expressed in this article are solely those of the authors and do not necessarily represent those of their affiliated organizations, or those of the publisher, the editors and the reviewers. Any product that may be evaluated in this article, or claim that may be made by its manufacturer, is not guaranteed or endorsed by the publisher.

## Supplementary Material

Supplementary Figure 1 Data flow through the experimental set-up. Created with BioRender.com.

Supplementary Table 1 Demographic characteristics of the study population. Numbers in brackets represent standard deviation. BMI, Body Mass Index. *N* = 8.

Supplementary Table 2 Mean grasp success rates (%) across tasks and feedback conditions. Numbers in brackets represent standard deviation. *N* = 8.

Supplementary Table 3 Mean energy expenditures (mV) across tasks and feedback conditions. Numbers in brackets represent standard deviation. *N* = 8.
