# Supplementary figures and images for: A Haptic Sleeve as a Method of Mechanotactile Feedback Restoration for Myoelectric Hand Prosthesis Users
Source: Front Rehabil Sci. 2022 Apr 25;3:806479. doi: 10.3389/fresc.2022.806479 (PMC9397846; doi:10.3389/fresc.2022.806479)

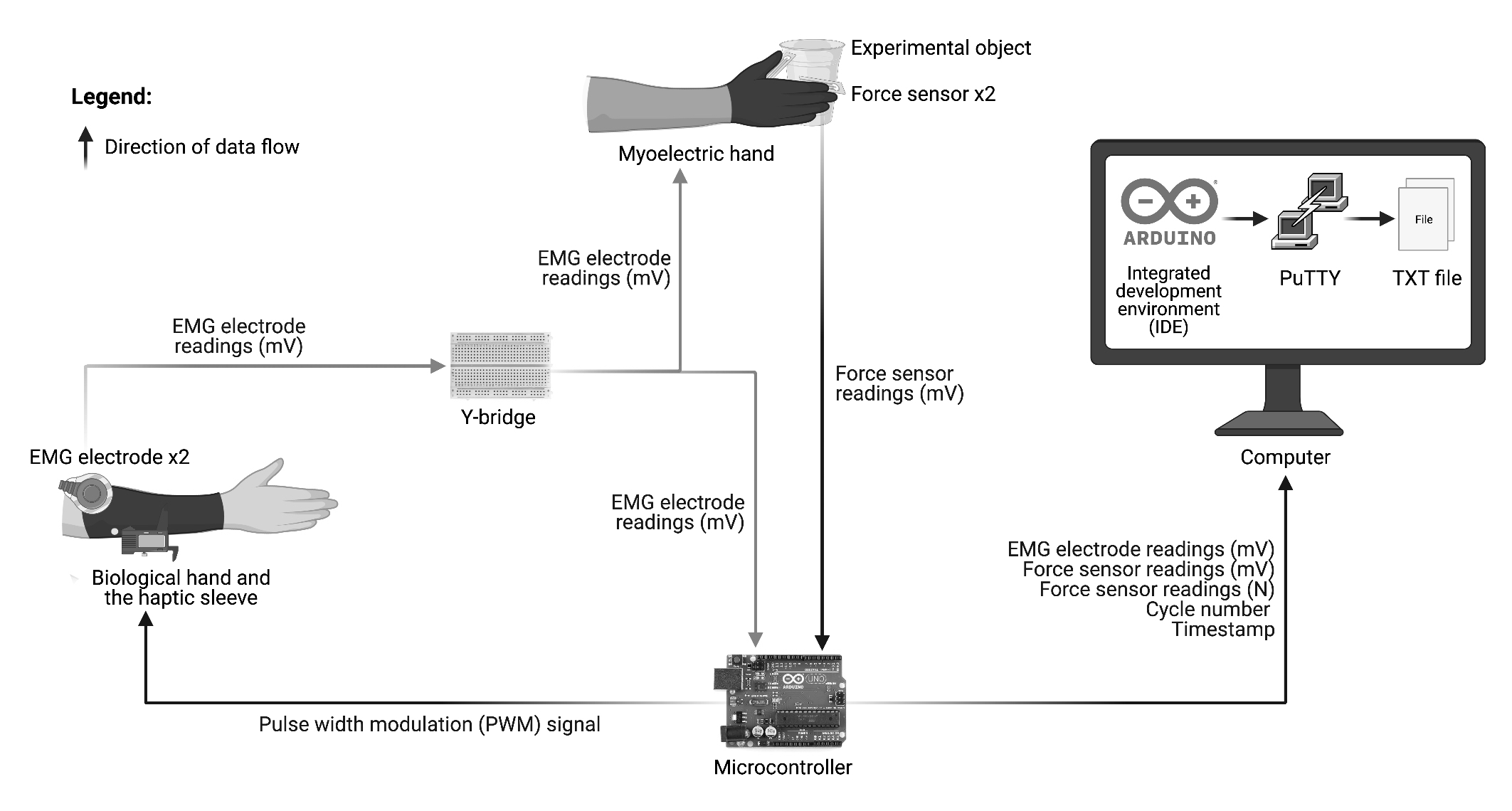

Supplement: Supplementary Figure 1 — Data flow through the experimental set-up. Created with BioRender.com. [file Image_1.JPEG]
