# Supplementary material for: A Haptic Sleeve as a Method of Mechanotactile Feedback Restoration for Myoelectric Hand Prosthesis Users
Source: Front Rehabil Sci. 2022 Apr 25;3:806479. doi: 10.3389/fresc.2022.806479 (PMC9397846; doi:10.3389/fresc.2022.806479)
Supplement: Supplementary Table 1 — Demographic characteristics of the study population. Numbers in brackets represent standard deviation. BMI, Body Mass Index. N = 8. [file Table_1.DOCX]

**Supplementary Table 1.** Demographic characteristics of the study population. Numbers in brackets represent standard deviation. BMI – Body Mass Index. N=8.

| Characteristic | Mean | Count | Range | Proportion (%) |
| --- | --- | --- | --- | --- |
| Age (years) | 33.3 (±10.4) | — | 21–55 | — |
| Sex  Male  Female | —  — | 5  3 | —  — | 62.5  37.5 |
| Handedness  Right-handed  Left-handed  Ambidextrous | —  —  — | 7  0  1 | —  —  — | 87.5  0.0  12.5 |
| BMI (kg/m^2^) | 25.3 (±5.9) | — | 16.7–33.2 | — |
| Forearm diameter (cm) | 8.7 (±1.3) | — | 6.7–10.2 | — |
