# Supplementary material for: A Haptic Sleeve as a Method of Mechanotactile Feedback Restoration for Myoelectric Hand Prosthesis Users
Source: Front Rehabil Sci. 2022 Apr 25;3:806479. doi: 10.3389/fresc.2022.806479 (PMC9397846; doi:10.3389/fresc.2022.806479)
Supplement: Supplementary Table 2 — Mean grasp success rates (%) across tasks and feedback conditions. Numbers in brackets represent standard deviation. N = 8. [file Table_2.DOCX]

**Supplementary Table 2.** Mean grasp success rates (%) across tasks and feedback conditions. Numbers in brackets represent standard deviation. N=8.

| Task | Feedback condition | Mean grasp success rate (%) | % difference with respective visual feedback condition | Significance of the difference (p-value) |
| --- | --- | --- | --- | --- |
| 1 | Visual | 70.0 (±10.7) | — | — |
|  | Visual + Haptic | 94.2 (±5.6) | +34.6 | <0.0001 |
|  | Haptic | 90.8 (±10.0) | +29.7 | <0.0001 |
| 2 | Visual | 69.2 (±11.2) | — | — |
|  | Visual + Haptic | 82.5 (±8.7) | +19.2 | 0.0057 |
|  | Haptic | 85.0 (±7.8) | +22.8 | 0.0015 |
| 3 | Visual | 87.5 (±9.0) | — | — |
|  | Visual + Haptic | 96.7 (±5.0) | +10.5 | 0.0170 |
|  | Haptic | 92.5 (±7.5) | +5.7 | 0.2260 |
