# Supplementary material for: A Haptic Sleeve as a Method of Mechanotactile Feedback Restoration for Myoelectric Hand Prosthesis Users
Source: Front Rehabil Sci. 2022 Apr 25;3:806479. doi: 10.3389/fresc.2022.806479 (PMC9397846; doi:10.3389/fresc.2022.806479)
Supplement: Supplementary Table 3 — Mean energy expenditures (mV) across tasks and feedback conditions. Numbers in brackets represent standard deviation. N = 8. [file Table_3.DOCX]

**Supplementary Table 3.** Mean energy expenditures (mV) across tasks and feedback conditions. Numbers in brackets represent standard deviation. N=8.

| Task | Feedback condition | Mean energy expenditure (mV) | % difference with respective visual feedback condition | Significance of the difference (p-value) |
| --- | --- | --- | --- | --- |
| 1 | Visual | 3033 (±1209) | — | — |
|  | Visual + Haptic | 2070 (±644) | -31.8 | <0.0001 |
|  | Haptic | 1920 (±660) | -36.7 | <0.0001 |
| 2 | Visual | 2998 (±1809) | — | — |
|  | Visual + Haptic | 3069 (±2032) | +2.4 | 0.7532 |
|  | Haptic | 2457 (±2068) | -18.1 | <0.0001 |
| 3 | Visual | 2186 (±657) | — | — |
|  | Visual + Haptic | 1997 (±559) | -8.7 | 0.0003 |
|  | Haptic | 1696 (±560) | -22.4 | <0.0001 |
